# Multidimensional Regulatory Network of *YAP1* Driving Malignant Progression in Esophageal Cancer: Molecular Mechanisms and Targeted Therapy: A Review

**DOI:** 10.32604/or.2026.073484

**Published:** 2026-04-22

**Authors:** Jun-Hui Chen, Si-Run Du, Chang Liu, Bei-Bei Liu, Hai-Ying Xu, Xin-Ying Ji, Bo Feng, Chun-Zheng Ma, Jun-Hui Guo

**Affiliations:** 1 The Second School of Clinical Medicine, Henan University of Traditional Chinese Medicine, Zhengzhou, China; 2 Henan Provincial Research Center of Engineering Technology for Nuclear Protein Medical Detection, Zhengzhou Health College, Zhengzhou, China; 3 Department of Oncology, The Second Affiliated Hospital of Henan University of Traditional Chinese Medicine, Zhengzhou, China

**Keywords:** Yes-associated protein 1, esophageal cancer, Hippo signaling pathway, molecular mechanisms, prognosis, targeted therapy

## Abstract

Esophageal cancer (EC) ranks among the most lethal gastrointestinal malignancies. Due to challenges in early diagnosis, molecular heterogeneity, and therapeutic resistance, patient prognosis remains extremely poor, necessitating the development of novel biomarkers and therapeutic targets. As a core effector of the Hippo signaling pathway, the potential significance of Yes-associated protein 1 (*YAP1*) has garnered increasing attention. This paper aims to systematically summarize the multi-omics research, molecular mechanisms, and preclinical/translational evidence for *YAP1*, covering its activation pathways, biological functions, clinical significance, and therapeutic strategies. We elucidated *YAP1*’s multidimensional regulatory network in EC, including Hippo-dependent and -independent mechanisms, cross-regulation with environmental risk factors, and its role in malignant phenotypes such as cell proliferation, apoptosis, epithelial-mesenchymal transition (EMT), and metastasis. The potential of *YAP1* as a diagnostic, prognostic, and predictive biomarker is evaluated, alongside summarizing its role in mediating chemotherapy, radiotherapy, and immune tolerance mechanisms, along with recent advances in targeted therapies. This provides a theoretical foundation for subsequent basic research and precision medicine translation. As a potential hub in the EC signaling network, it is considered to play a key role in driving tumor progression and treatment resistance through multiple pathways. Targeting *YAP1* holds broad clinical promise but faces challenges including functional duality, subtype heterogeneity, and complex resistance mechanisms. Future efforts should focus on developing highly selective inhibitors, integrating multi-omics technologies and innovative models to advance clinical translation and provide new strategies for precision treatment of EC patients.

## 1 Introduction

According to the latest global cancer statistics, esophageal cancer (EC)is a highly prevalent gastrointestinal malignant tumor worldwide, with the eleventh highest incidence rate and the seventh highest mortality rate [1]. Despite significant advances in diagnostic and therapeutic techniques, the 5-year survival rate of EC patients is still less than 20%, and the treatment of EC faces multiple challenges; thus, there is an urgent need to explore new therapeutic targets and strategies.

In recent years, Yes-associated protein 1 (*YAP1*)has emerged playing a pivotal role as a core effector molecule in the Hippo signaling pathway. The aberrant activation of *YAP1* in regulating malignant tumor progression, especially in EC, is closely related to proliferation, metastasis, differentiation, apoptosis and microenvironmental remodeling. Numerous excellent articles on *YAP1* have emerged. For example, Cinar et al. systematically summarized the classic components and regulatory mechanisms of the Hippo pathway [2], while the Maehama team brilliantly outlined the molecular regulation and clinical application potential of Hippo–*YAP1* signaling in squamous cell carcinoma [3]. Additionally, some studies have begun exploring *YAP1’*s potential as a target for research [4]. However, existing literature predominantly focuses on single dimensions and exhibits several limitations: ① absence of environmental carcinogenic factors; ② insufficient pathway integration; ③ missing clinical translation pathways; ④ lack of spatial structural maps to visualize regulatory networks.

**Figure 1:**
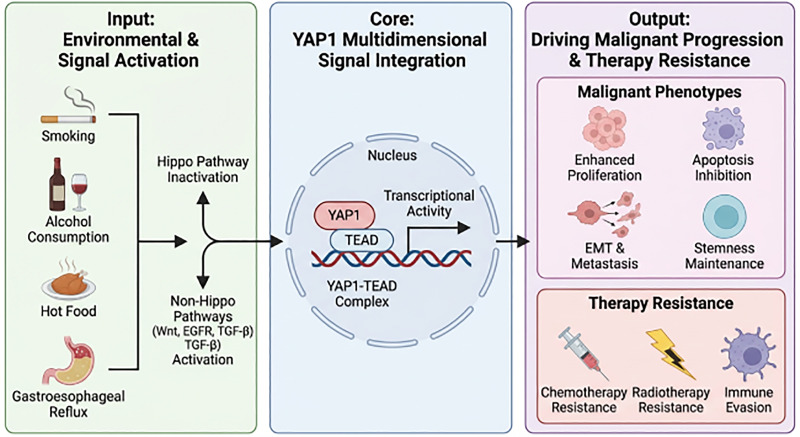
Network model of progression *YAP1* as a multidimensional regulatory hub driving EC. This schematic diagram summarizes the core functional framework of *YAP1* in EC. The left panel demonstrates that multiple environmental risk factors (e.g., smoking, alcohol consumption, hot food intake, gastroesophageal reflux) collectively activate *YAP1* through Hippo-dependent or independent signaling pathways (e.g., Wnt/β-catenin, EGFR, TGF-β). Activated *YAP1* translocates to the nucleus, forming a stable complex with the TEAD transcription factor (core hub) to remodel downstream gene transcription programs. The right panel outputs demonstrate that the *YAP1*-TEAD complex comprehensively drives EC progression and recurrence by: Driving key malignant biological phenotypes including sustained proliferation, apoptosis suppression, EMT and metastasis, and maintenance of stem cell properties; Simultaneously mediating multimodal resistance to chemotherapy, radiotherapy, and immunotherapy. Abb: esophageal cancer (EC); Yes-associated protein 1 (*YAP1*); TEA domain (TEAD); epithelial-mesenchymal transition (EMT); epidermal growth factor receptor (EGFR); transforming growth factor-beta (TGF-β).

This review aims to fill the aforementioned gap by proposing and exploring a trinity-based multidimensional regulatory network hypothesis encompassing “carcinogenic mechanisms–clinical significance–targeted therapy.” This paper systematically integrates: ① the molecular interaction network between environmental carcinogens and *YAP1*; ② bidirectional regulatory mechanisms in Hippo-dependent and -independent pathways; ③ a complete evidence chain from fundamental mechanisms to clinical translation, particularly the relationship between therapeutic resistance and targeted strategies; ④ analysis of existing limitations using visual structural diagrams to propose future research directions. The abstract diagram is presented in Fig. 1.

## 2 Theoretical Background

### 2.1 Epidemiology and Molecular Characteristics of EC

EC is a highly invasive and lethal gastrointestinal malignancy, categorized into two subtypes: esophageal squamous cell carcinoma (ESCC) and esophageal adenocarcinoma (EAC). Most patients lack specific symptoms in the early stages, leading to diagnosis at an advanced stage. Furthermore, significant molecular heterogeneity and drug resistance increase the risk of recurrence or metastasis [5]. Current treatments still offer limited benefits, making it imperative to identify new targets and elucidate their molecular mechanisms to improve survival.

The epidemiological characteristics of EC exhibit significant regional differences: ESCC is prevalent in Asia and associated with factors such as smoking and alcohol consumption, while EAC is common in Western populations and linked to gastroesophageal reflux disease and Barrett’s esophagus [6]. At the molecular level, the two exhibit distinct genetic mutation profiles. ESCC frequently exhibits mutations in TP53, CDKN2A, and PIK3CA [7]; it is also associated with high genomic instability, leading to rapid progression and poor prognosis [8]. In contrast, EAC predominantly exhibits mutations in TP53, ARID1A, and ERBB2 [9]. This disparity also influences treatment response: ESCC shows limited response to chemotherapy and immunotherapy, whereas EAC demonstrates greater sensitivity to targeted therapies [10,11]. These differences suggest that *YAP1* may function through distinct mechanisms and modes of action in the two subtypes.

### 2.2 Basis of the Hippo–YAP1 Signaling Pathway

The Hippo signaling pathway is a highly conserved signal transduction pathway that regulates downstream effector *YAP1* through phosphorylation, participating in organ development and stem cell growth while being closely associated with various malignant tumors. The core mechanism of this pathway involves, for example, *in vitro* cellular experiments demonstrating that the OTU deubiquitinating enzyme 2 (OTUB2) stabilizes *YAP1*/TAZ proteins. Knocking down OTUB2 inhibits the proliferation and migration of ESCC cells [12].

In normal cells, core kinases of the Hippo pathway (such as MST1/2 and LATS1/2) restrict *YAP1* activity, preventing abnormal proliferation. However, in EC cells, dysregulation of the Hippo pathway abnormally activates *YAP1*, potentially promoting tumorigenesis and progression [13]. Furthermore, preclinical studies indicate that *YAP1* activity may correlate with the tumor microenvironment (such as angiogenesis and immune infiltration), enhancing tumor cell survival and invasiveness [14] When Hippo signaling is inhibited, *YAP1*/TAZ undergoes dephosphorylation and translocates to the nucleus, where it binds to other transcription factors. This may induce cell proliferation, suppress apoptosis, and drive malignant transformation [15].

### 2.3 Structural and Functional Characteristics of YAP1

*YAP1* is located on chromosome 11q13, with a molecular weight of approximately 65 kDa. It comprises an N-terminal proline-rich region, a C-terminal transcription activation domain, a WW domain, and a PDZ-binding domain, which collectively mediate protein interactions and determine its functional specificity [16]. The WW domain specifically binds target proteins to trigger cell proliferation and survival signals [17]. As a transcriptional coactivator, *YAP1* does not directly bind DNA but primarily regulates gene expression by interacting with transcription factors. Its nuclear transport and activity are modulated by multiple signaling pathways [18,19].

The TEA domain (TEAD) transcription factor family represents one of *YAP1’*s primary targets. By binding to *YAP1* via its TEA domain, TEAD forms a transcription activation complex that promotes cell growth and differentiation. TEAD family members exhibit differences in tissue specificity and biological functions, suggesting that *YAP1*-TEAD complexes may perform distinct roles across various cell types. For example, in certain cancer models, *YAP1*-TEAD may enhance tumor cell proliferation and survival [20,21]. *YAP1*-TEAD is extensively involved in multiple biological processes, including organ development, tissue homeostasis, cell proliferation, migration, epithelial-mesenchymal transition (EMT), and apoptosis regulation, suggesting its potential value as a therapeutic target in tumor treatment [22].

### 2.4 Role of YAP1 in Various Cancers

*YAP1* function exhibits cancer type and context dependence, exhibiting both oncogenic and tumor-suppressive roles. Most studies indicate that *YAP1* may act as an oncogene promoting tumor progression. Based on *in vitro* and animal model evidence, *YAP1* influences the growth, proliferation, invasion, metastasis, and chemotherapy resistance of various tumors, such as non-small cell lung cancer [23], prostate cancer [24], and liver cancer [25].

However, some studies suggest *YAP1* may also exert tumor-suppressive effects in cancer. In breast cancer, *YAP1* knockdown promotes tumor cell migration and proliferation in both *in vivo* and *in vitro* experiments [26]. Following liver resection, activation of *YAP1* in hepatocytes suppresses colorectal cancer liver metastasis by regulating gene expression related to glutamine metabolism, thereby inducing glutamine deprivation in tumor cells [27]. *YAP1* may also promote ferroptosis and inhibit cell growth [28].

## 3 Current Research Status of YAP1 in EC

### 3.1 Abnormal Expression in EC and Clinical Associations

The high expression of *YAP1* in EC and its association with clinical prognosis have become key entry points for translational research. While studies on *YAP1* in ESCC are relatively extensive and well-established, evidence in EAC remains in its early stages. In both ESCC and EAC, *YAP1* mRNA and protein levels are elevated compared to adjacent normal tissue. In ESCC, overexpression correlates with increased invasiveness and poor prognosis, whereas evidence in EAC is limited and primarily derived from small samples or indirect inferences [29]. Retrospective clinical-pathological analyses indicate a negative correlation between *YAP1* expression and patient survival in ESCC, while no significant association has been observed in EAC [30]. Multicancer bioinformatics analysis revealed that high *YAP1* expression correlates with resistance to anti-PD-1 therapy, suggesting its potential as a predictive marker for immunotherapy efficacy; however, this finding is inferred from public databases and lacks validation in clinical cohorts [31]. Regarding pathological characteristics, *YAP1* expression levels negatively correlate with tumor differentiation, and the lymph node metastasis rate is significantly higher in the high-expression group compared to the low-expression group, showing a positive correlation with TNM staging [32].

However, some studies suggest potential stage heterogeneity in the prognostic role of *YAP1* in EC, potentially due to differences in detection methods, analysis endpoints (overall survival [OS] vs. disease-free survival [DFS]), and patient treatment histories, leading to inconsistent results. Kuo et al. found that patients with high *YAP1* expression had longer OS, potentially serving as an independent prognostic protective factor [33]. Analysis based on the TCGA database also showed that high *YAP1* expression in ESCC correlates with better patient prognosis, though this conclusion remains unvalidated in EAC samples [34]. Among long-term survivors of ESCC, *YAP1*-positive patients exhibited significantly prolonged DFS and OS [35]. This protective effect may be related to *YAP1* activation of the PML pathway and promotion of ferroptosis, but the specific mechanism requires further elucidation.

In summary, *YAP1* exhibits complex functionality in EC, potentially influenced by multiple factors including tumor stage, molecular subtype, and microenvironment. However, existing evidence largely relies on retrospective analyses with limited sample sizes and inconsistent follow-up and treatment backgrounds. Notably, the lack of multicenter prospective validation for EAC data restricts the clinical application of *YAP1* as a universal biomarker.

### 3.2 Dual Pathways of YAP1 Oncogenesis: Hippo-Dependent and Hippo-Independent Pathways

Abnormal activation of *YAP1* may serve as a core driver of EC malignant progression. Its activation mechanisms primarily encompass both classical Hippo pathway-dependent approaches and multiple non-Hippo pathway-dependent approaches, collectively forming a highly complex regulatory network.

#### 3.2.1 Hippo-Dependent Oncogenic Mechanisms

**Figure 2:**
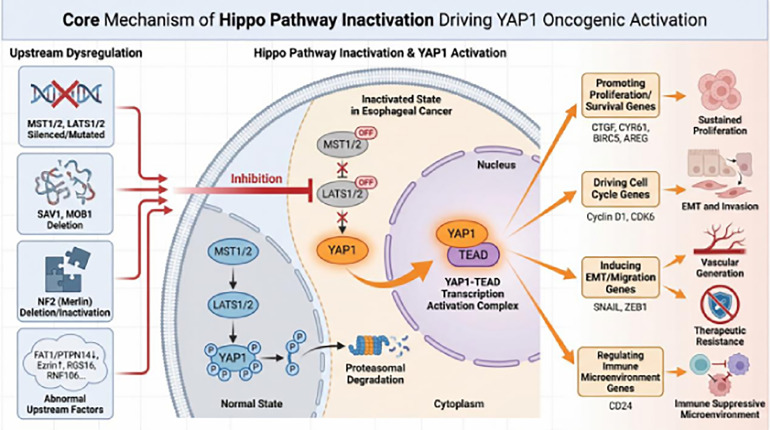
Core mechanism of *YAP1* oncogenic activation driven by Hippo pathway inactivation. This figure summarizes the key molecular events leading to abnormal *YAP1* activation via Hippo-dependent pathways in EC. The upper left panel lists upstream events causing Hippo signaling inactivation, including silencing or mutation of core kinases (MST1/2, LATS1/2), loss of scaffolding proteins (SAV1, MOB1), loss of function of the tumor suppressor NF2, and abnormalities in other regulatory factors (e.g., FAT1, Ezrin). These events collectively inhibit the activity of the Hippo kinase cascade (red inhibitory symbols). The central cellular schematic compares the normal state with the abnormal state in ECs. Under normal conditions, the active Hippo pathway promotes phosphorylation of *YAP1* (p-*YAP1*) and its retention in the cytoplasm for degradation. When the pathway is inactivated, *YAP1* undergoes dephosphorylation and translocates to the nucleus. Within the nucleus, dephosphorylated *YAP1* binds to TEAD transcription factors to form a stable transcription activation complex. This complex subsequently initiates transcription of a series of downstream oncogenic target genes involved in cell proliferation, cell cycle progression, EMT, and immune regulation. Ultimately, the activation of these gene programs collectively drives key malignant phenotypes of EC, including sustained proliferation, invasion and metastasis, angiogenesis, therapeutic resistance, and the formation of an immunosuppressive microenvironment. Abb: phosphorylation of *YAP1* (p-*YAP1*); mammalian STE20-like kinase (MST); large tumor suppressor kinase (LATS); salvador homolog 1 (SAV1); MOB kinase activator (MOB); neurofibromin 2 (NF2); FAT atypical cadherin (FAT); protein tyrosine phosphatase, non-receptor type (PTPN);regulator of G-protein signaling (RGS); RING finger protein (RNF);connective tissue growth factor (CTGF); cysteine-rich angiogenic inducer 61 (CYR61); baculoviral IAP repeat-containing protein (BIRC); amphiregulin (AREG); cyclin-dependent kinase (CDK); snail family transcriptional repressor (SNAIL); zinc finger E-box-binding homeobox (ZEB); cluster of differentiation (CD).

The Hippo signaling pathway is the core pathway regulating *YAP1* activity, Its dysregulation has been associated with malignant progression in multiple preclinical studies (as shown in Fig. 2).

Mechanisms of Inactivation of Core Components

The normal function of the Hippo pathway depends on the activation of its core kinase cascade, with MST1/2 and LATS1/2 potentially serving as key drivers of EC malignant progression. *In vitro* cell experiments indicate that silencing or mutating MST1/2 may disrupt the inhibition of *YAP1*, activate downstream target genes, and promote EC proliferation and migration. In multiple tumor animal models, LATS1/2 acts as a downstream effector; its inactivation may release negative regulation of *YAP1* [36]. As scaffolding proteins of the Hippo pathway, functional loss of SAV1 and MOB1 may lead to *YAP1* activation, promoting EC proliferation and invasion, and correlating with poor patient prognosis [37]. Clinical analysis indicates that the NF2 gene, a key inhibitor in the Hippo pathway, activates MST1/2 to suppress *YAP1* activity and is associated with EC progression and poor prognosis [38].

Furthermore, loss of the upstream regulator FAT1/PTPN14 may induce LATS2 degradation and *YAP1* activation, promoting ESCC progression and cisplatin resistance [13]. Ezrin also serves as a potential upstream regulator of *YAP1*, exhibiting high expression in ESCC and showing positive correlation with *YAP1* levels. Its downregulation may inhibit ESCC cell proliferation, migration, and invasion [14]. RGS16 may disrupt the interaction between MST1 and LATS1, thereby inhibiting *YAP1* phosphorylation, promoting its nuclear translocation and transcriptional activity, and ultimately driving ESCC progression [39]. Molecular biology experiments suggest that RNF106 may inhibit *YAP1* phosphorylation and activate its oncogenic functions by promoting K48-linked ubiquitination and degradation of LATS2, thereby driving ESCC progression. This pathway remains a hypothesis requiring further clinical validation [40].

The above evidence suggests that functional loss of core Hippo components may be a prerequisite for *YAP1* activation, but its specific role across different subtypes and disease stages requires further demonstration.

*YAP1*-TEAD Downstream Target Gene Network

Upon nuclear localization, *YAP1* forms a transcription activation complex by binding to TEAD, thereby regulating downstream gene expression. It may play a crucial role in processes such as cell proliferation, migration, survival, and extracellular matrix (ECM) remodeling. However, current evidence remains limited to preclinical studies, and validation in clinical samples with spatiotemporal dynamics requires further investigation.

CTGF and CYR61 are primary target genes of the *YAP1*-TEAD pathway. Multi-cancer studies indicate that *YAP1* may promote ECM deposition and tumor microenvironment remodeling by regulating CTGF and CYR61 expression [41,42]. *In vitro* evidence suggests *YAP1*-TEAD may modulate the expression of AREG, an EGFR ligand, thereby influencing tumor cell proliferation and migration [43,44]. *YAP1*-TEAD activates cell cycle-related genes such as BIRC5 and CDC20, accelerating cell cycle progression and driving tumor malignancy [45].

Other regulatory factors, such as SQLE gene amplification, may promote YAP nuclear accumulation by interacting with Vinculin protein, thereby activating Hippo downstream target genes and potentially contributing to ESCC development [46]. Overexpression of C12orf59 may induce YAP dephosphorylation and nuclear translocation, thereby activating EMT-related genes and accelerating ESCC metastasis [47]. Knocking down AJUBA reduces *YAP1* and TAZ expression, inhibits their nuclear localization, and leads to ESCC cell cycle arrest, weakened cloning ability, and decreased migration and invasion capacity [48]. In carcinogen-induced ESCC animal models, targeting *YAP1* effectively inhibited cell proliferation and tumor growth *in vivo* and *in vitro*, revealing that excessive activation of the Hippo-*YAP1* axis may be a key driver of ESCC progression [49]. Dysregulation of the Hippo-*YAP1* pathway has been observed in molecular analyses of multiple squamous cell carcinomas. *YAP1* overexpression correlates positively with ESCC histological grade, clinical stage, and lymph node metastasis, suggesting its nuclear accumulation drives tumor initiation and progression [3].

#### 3.2.2 Regulatory Mechanisms of the Non-Hippo-Dependent Pathway

**Figure 3:**
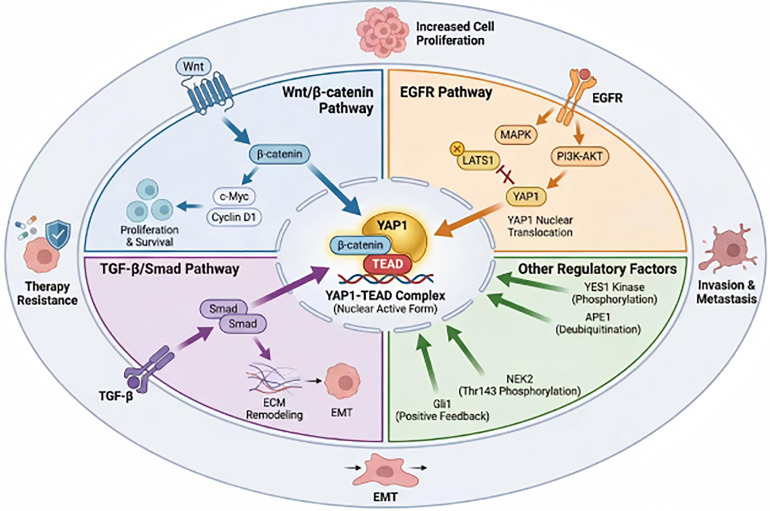
Molecular mechanism of *YAP1* regulating EC malignant progression via non-Hippo pathways. This diagram illustrates the core mechanism by which *YAP1* is activated through multiple non-Hippo pathways in EC. At the center is the nuclear *YAP1*-TEAD complex, with peripheral components including: ① In the Wnt/β-catenin pathway, *YAP1* synergistically activates c-Myc and Cyclin D1 with β-catenin, promoting cell proliferation and survival; ② The EGFR pathway inhibits LATS1 kinase via MAPK and PI3K-AKT signaling, facilitating *YAP1* nuclear translocation and enhancing transcriptional activity; ③ In the TGF-β/Smad pathway, direct interaction between Smad proteins and *YAP1* induces ECM remodeling and EMT; ④ Other regulatory factors such as YES1 kinase, APE1, NEK2, and Gli1 activate *YAP1* through phosphorylation, deubiquitination, or transcriptional feedback. These signals collectively drive proliferation, invasion, metastasis, and treatment resistance in EC cells, forming a complex non-Hippo-dependent regulatory network. Abb: extracellular matrix (ECM); SMAD family member (Smad); cellular myelocytomatosis oncogene (c-Myc); mitogen-activated protein kinase (MAPK); phosphoinositide 3-kinase (PI3K); protein kinase B (AKT); apurinic/apyrimidinic endonuclease 1 (APE1); YES proto-oncogene 1, Src family tyrosine kinase (YES1); never in mitosis gene A-related kinase (NEK); GLI family zinc finger (Gli); Wingless-related integration site (Wnt).

The activity of *YAP1* is also directly regulated by multiple key signaling pathways, including Wnt/β-catenin, EGFR, and TGF-β/Smad. These pathways form complex positive feedback and synergistic activation networks with *YAP1*, potentially contributing collectively to EC progression (as shown in Fig. 3).

Interaction with the Wnt/β-Catenin Pathway

In the malignant progression of EC, the interaction between *YAP1* and the Wnt/β-catenin pathway may exert significant regulatory effects, involving the co-regulation of multiple target genes. *In vitro* cellular experiments demonstrate that when *YAP1* is highly expressed, β-catenin expression also significantly increases, jointly activating target genes c-Myc and Cyclin D1. This may promote cell proliferation and survival, playing a crucial role in EC progression [50,51]. Furthermore, *YAP1* may enhance Wnt signaling by stabilizing β-catenin in the nucleus [52]. *YAP1* and the Wnt/β-catenin pathway may also exert synergistic effects in inducing EMT, such as upregulating N-cadherin and vimentin while simultaneously suppressing E-cadherin [53,54].

Interaction with the EGFR Signaling Pathway

EGFR activates downstream signaling pathways by binding to EC ligands, thereby promoting cell growth, survival, and metastasis, and is associated with the malignant progression of EC. *In vitro* data support the positive feedback loop hypothesis, suggesting that *YAP1* may upregulate EGFR expression; conversely, activated EGFR may promote *YAP1* nuclear translocation by inhibiting LATS1 phosphorylation, jointly contributing to ESCC progression [55]. EGFR activation can also promote *YAP1* activation via the SRC signaling pathway, leading to increased resistance of tumor cells to EGFR inhibitors [56].

MAPK and PI3K-AKT, as major downstream effectors of EGFR signaling, participate in *YAP1* regulation. MAPK promotes *YAP1* phosphorylation by inhibiting its negative regulator DUSP1, thereby enhancing its transcriptional activity [57]. Conversely, the PI3K-AKT pathway accelerates *YAP1*-mediated tumorigenesis by facilitating its nuclear translocation and strengthening its binding capacity to TEAD [58].

Interaction with the TGF-β/Smad Pathway

*YAP1* exhibits complex interactions with the TGF-β signaling pathway and may play a crucial role in cell proliferation, differentiation, and ECM remodeling. *In vitro* protein interaction studies reveal a physical interaction between Smad proteins and *YAP1*, which enhances *YAP1’*s transcriptional activity and is essential for cellular functions during embryonic development and tissue repair [59]. TGF-β stimulation induces *YAP1* activation, thereby promoting the expression of EMT-associated genes such as N-cadherin and vimentin. This facilitates morphological changes in tumor cells and enhances their migratory capacity [60]. Within the tumor microenvironment, *YAP1* influences downstream effects of the TGF-β signaling pathway. By regulating the expression of matrix-associated genes, it enhances cell-matrix interactions, thereby promoting tumor cell survival and migration [61].

However, given the complexity of TGF-β signaling, the precise role and regulatory mechanisms of *YAP1* within its network may be highly dependent on the tumor microenvironment and disease stage, presenting challenges for future research.

Interactions with Other Key Pathways and Factors

Other key regulatory factors precisely modulate *YAP1* through diverse mechanisms.

Clinical studies demonstrate that YES1 kinase regulates *YAP1* nuclear-cytoplasmic shuttling through phosphorylation modification; YES1 inhibitors effectively block *YAP1* nuclear localization and inhibit tumor growth [62]. *In vivo* mouse studies indicate that targeting Ape1 suppresses *YAP1* activity, delays tumor growth in models, and inhibits tumor stem cell properties and EAC progression [63].

Regarding cytokine-receptor signaling, in ESCC, autocrine leukemia inhibitory factor activates the SFK-YAP pathway, driving ESCC proliferation, migration, invasion, and stem cell properties [64]. Inhibition of FGFR2 induces NF2 ubiquitination and degradation, activating *YAP1* to transcribe RIP1 and MLKL and induce necrotic apoptosis in ESCC [65].

In terms of epigenetic and transcriptional regulation, knockdown of JMJD1C inhibits *YAP1* expression and cell proliferation, while overexpression of *YAP1* reverses this effect [66].

Regarding post-translational modifications, NEK2 may stabilize *YAP1* protein and promote EMT by phosphorylating *YAP1* at Thr-143 to block its ubiquitin-mediated degradation, thereby driving ESCC progression [67].

In cross-pathway transcriptional feedback, the Hedgehog pathway transcription factor Gli1 forms a positive feedback loop with *YAP1*: Gli1 upregulates *YAP1* via a LATS1-independent mechanism, while *YAP1* activates Gli1 expression through the PI3K/AKT pathway [30].

Regarding independent transcription targets, *YAP1*/TEAD4 directly activates KIF4A transcription, driving ESCC proliferation, migration, and apoptosis inhibition, thereby providing a potential combined therapeutic target for ESCC [68].

In summary, these complex pathway interactions demonstrate that *YAP1* serves as a crucial hub in the EC signaling network by integrating the Hippo pathway with other signaling pathways. This provides a theoretical foundation for developing precision therapeutic strategies targeting *YAP1* or its upstream/downstream molecules (as shown in Table 1). However, whether these molecular-level interactions directly drive alterations in EC malignancy and their relevance *in vivo* remain unresolved scientific questions, presenting significant challenges and opportunities.

**Table 1:** Hippo-dependent and -independent oncogenic mechanisms of *YAP1* in EC.

Mechanism Type	Key Pathway/Factor	Primary Function/Effect	Reference
**Hippo-dependent**	Inactivation of MST1/2 and LATS1/2	Kinase inactivation, *YAP1* dephosphorylation and nuclear translocation, promoting proliferation and invasion	[36]
	SAV1, MOB1, NF2 deletion	Loss of scaffold or tumor suppressor protein function leads to Hippo pathway inactivation and *YAP1* activation	[37,38]
**Hippo-independent**	Wnt/β-catenin pathway	*YAP1* synergistically activates c-Myc and Cyclin D1 with β-catenin	[50,51]
	EGFR pathway	Inhibits LATS1, promotes *YAP1* nuclear translocation and transcriptional activity	[55,58]
	TGF-β/Smad pathway	Smad interacts with *YAP1*, inducing EMT and metastasis	[59–61]
	YES1, APE1, etc.	Activate *YAP1* through phosphorylation, deubiquitination, and other mechanisms	[62,63,68]

Note: esophageal cancer (EC); Yes-associated protein 1 (*YAP1*); mammalian STE20-like kinase (MST); large tumor suppressor kinase (LATS); salvador homolog 1 (SAV1); MOB kinase activator (MOB); neurofibromin 2 (NF2); epidermal growth factor receptor (EGFR); transforming growth factor-beta (TGF-β); YES proto-oncogene 1, Src family tyrosine kinase (YES1); apurinic/apyrimidinic endonuclease 1 (APE1); epithelial-mesenchymal transition (EMT).

### 3.3 Molecular Interactions between YAP1 and Environmental Risk Factors in EC Development

Given that the occurrence of EC is associated with multiple environmental risk factors, *YAP1* may play a central role in the process where environmental carcinogens promote the development and progression of EC.

In gastroesophageal reflux disease, APE1 interferes with the ubiquitination and degradation of *YAP1* through its redox function, potentially promoting the progression from Barrett’s esophagus to EAC [63]. Chronic inflammation induced by hot food and mechanical injury may trigger *YAP1* nuclear translocation and upregulate EMT, contributing to EC development [69]. Furthermore, alcohol and tobacco extracts synergistically inhibit the Hippo pathway, activating the *YAP1*/TAZ signaling axis, which may subsequently impede pyroptosis and drive malignant transformation of paraneoplastic tissues [70]. Clinical analyses reveal an association between *YAP1* expression and patient smoking history. Nicotine induces *YAP1* dephosphorylation and nuclear translocation via the nAChRs-PKC axis, promoting ESCC proliferation, migration, and apoptosis inhibition [71].

**Figure 4:**
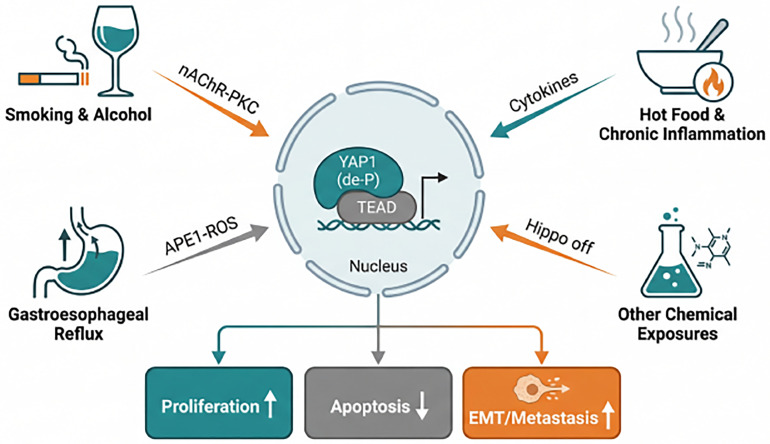
Environmental risk factors drive EC development by activating *YAP1*. Major environmental risk factors (top left: smoking/alcohol; top right: hot food or chronic inflammation; bottom left: gastroesophageal reflux; bottom right: other chemical exposures) inhibit Hippo or stabilize *YAP1* via their respective signaling axes, thereby promoting the activation of the *YAP1*-TEAD complex within the cell nucleus. The activated complex upregulates proliferation genes, suppresses apoptosis, and induces EMT, thereby driving the initiation and progression of EC.

In summary, *YAP1* may serve as a pivotal node in the synergistic action of multiple environmental carcinogenic factors, with mechanisms involving the inflammatory microenvironment, epigenetic regulation, and the interplay of diverse signaling pathways (as shown in Fig. 4 and Table 2).

**Table 2:** Molecular interactions between *YAP1* and environmental risk factors in the development of EC.

Environmental Factors	Mechanism of Action	Biological Effects	References
**Smoking (Nicotine)**	Induces *YAP1* dephosphorylation and nuclear translocation via the nAChRs-PKC axis	Promotes ESCC proliferation, migration, and inhibits apoptosis	[71]
**Alcohol**	Inhibits the Hippo pathway, activates *YAP1*/TAZ	Inhibits pyroptosis, promotes malignant transformation of paracellular tissues	[70]
**Thermal/mechanical injury**	Induces *YAP1* nuclear translocation, enhancing transcriptional activity	Triggering EMT and promoting tumor initiation	[69]
**Gastroesophageal reflux disease**	APE1 upregulation interferes with *YAP1* ubiquitination and degradation, enhancing its stability	Promotes progression from Barrett’s esophagus to EAC	[63]

Note: esophageal squamous cell carcinoma (ESCC); esophageal adenocarcinoma (EAC); transcriptional coactivator with PDZ-binding motif (TAZ).

### 3.4 YAP1-Mediated Oncogenic Biological Functions

Increasing evidence suggests that activated *YAP1* may promote the malignant phenotype of EC by regulating a series of key downstream target genes and biological processes. The oncogenic functions include the following:

#### 3.4.1 Driving Proliferation and Inhibiting Apoptosis

In EC progression, *YAP1* may maintain cellular survival advantage and accelerate tumor growth by activating proliferation genes and inhibiting apoptosis.

*YAP1* may promote EAC cell proliferation by regulating cell cycle modulators, thereby influencing the transition between G1/S and G2/M phases [63]. JMJD1C promotes ESCC growth by upregulating *YAP1* through reducing H3K9me2 modification in the *YAP1* promoter region [66]. Gli1 interacts with *YAP1* to promote ESCC cell proliferation and migration; both are highly expressed and predict poor prognosis [30]. *In vitro* experiments and animal model studies indicate that nuclear accumulation of *YAP1* activates CD24 transcription or drives EC cells to evade macrophage phagocytosis, maintaining cancer cell survival advantages [72]. The *YAP1*-TEAD complex upregulates IRS2 expression via the JNK/c-Jun pathway, accelerating cell cycle progression [73]. TEAD4 forms a complex with *YAP1*, jointly promoting ESCC cell proliferation, migration, and invasion; inhibiting TEAD4-*YAP1* interaction effectively blocks these malignant phenotypes [74].

Regarding apoptosis regulation, *YAP1* inhibits mitochondrial apoptosis by balancing the expression of key apoptotic modulators such as Bcl-2/Bax, thereby enhancing EC cell survival [32]. Liu et al. found that SQLE metabolites bind to Vinculin, promoting *YAP1* nuclear translocation and activating anti-apoptotic genes [46].

#### 3.4.2 Promotion of EMT, Invasion and Metastasis

EMT is a critical step in the development of local invasion and distant metastasis in EC. *YAP1* plays a significant role in promoting tumor invasion and metastasis, or acts as one of the core regulatory factors of EMT, enhancing the invasive and metastatic capabilities of EC cells.

Pan-cancer multi-omics studies indicate that patients in the subgroup exhibiting Hippo-*YAP1* immune co-activation show the poorest prognosis. In organ model studies, USP36 stabilizes YAP protein through deubiquitination, enhancing its transcriptional activity and thereby driving EMT [16]. Sun et al. discovered that RNF106 degrades LATS2 via K48-linked ubiquitination, relieving its inhibitory phosphorylation of YAP. This promotes expression of key EMT proteins and accelerates ESCC invasion and metastasis [40]. Xu et al. confirmed that C12orf59 overexpression leads to YAP dephosphorylation and nuclear localization, activating EMT transcription factors such as SNAIL [47]. Overexpressed *YAP1* promotes EMT, a process potentially linked to enhanced invasiveness and improved circulatory survival [67]. This mechanism involves *YAP1* regulating transcription factors such as Snail, ZEB1, and Twist to drive cellular EMT, thereby enhancing ESCC cell migration [75]. GPRC5A promotes LATS1 ubiquitination and degradation by binding to the E3 ubiquitin ligase WWP1, thereby releasing inhibition of *YAP1*. This mediates an EMT-like phenotype and drives lung metastasis in ESCC [76]. Furthermore, *in vitro* studies suggest a potential positive feedback loop between *YAP1* and EMT: the EMT process can activate YAP through multiple mechanisms, while activated YAP further intensifies the EMT phenotype [77].

These preclinical studies provide new insights into the metastatic mechanisms of EC and lay the foundation for developing novel targeted therapeutic strategies. However, their clinical translational value requires further validation.

### 3.5 Potential of YAP1 as a Diagnostic, Prognostic and Predictive Treatment for EC

*YAP1* is widely overexpressed inESCC and correlates with malignant tumor phenotypes, demonstrating significant potential in histological diagnosis, prognostic assessment, and prediction of treatment response.

In terms of diagnosis, immunohistochemical analysis indicates that *YAP1* is highly expressed in ESCC tissues and positively correlates with tumor size, differentiation grade, depth of invasion, and lymph node metastasis, suggesting *YAP1* may serve as a prognostic marker for clinical risk stratification [29]. Immunohistochemistry confirms co-expression of Ezrin and *YAP1* in cancerous tissues, both associated with poor prognosis, making them potential indicators for ESCC progression assessment [14]. Liquid biopsy studies suggest activation of the *YAP1*/EGFR axis correlates with treatment resistance, though its value requires further prospective validation [55].

Regarding prognostic assessment, immunohistochemical and transcriptomic analyses based on the TCGA-ESCC cohort and GEO database revealed that patients with high nuclear localization of *YAP1* exhibited shorter overall survival (OS) and progression-free survival (PFS), particularly in subgroups receiving platinum-based chemotherapy or chemoradiotherapy [65]. Multi-omics analysis revealed that the long non-coding RNA KTN1-AS1 activates the *YAP1* signaling pathway by regulating miR-885-5p and STRN3 expression, thereby promoting tumor progression [78].

Furthermore, *YAP1* interacts with the immune microenvironment, where its expression level influences CD4^+^ T-cell infiltration and may serve as a predictor of immunotherapy response [79]. A multicenter real-world study confirmed that *YAP1*-positive ESCC patients had a 3-year OS rate of 38.6% after standard chemoradiotherapy, significantly lower than the 57.4% in the negative group, and was associated with a higher risk of local recurrence [80].

Collectively, findings ranging from histopathological associations to multicenter real-world data indicate *YAP1’*s potential as a robust independent prognostic and predictive factor. These discoveries provide critical molecular evidence for clinical risk stratification, prognosis assessment, and personalized treatment guidance. However, variations in genetic backgrounds and clinical representativeness across different models may limit the applicability of these conclusions when extrapolated to the real world.

### 3.6 The Significance of YAP1 in the Treatment of EC: Therapeutic Resistance Mechanisms and Targeted Intervention Strategies

**Figure 5:**
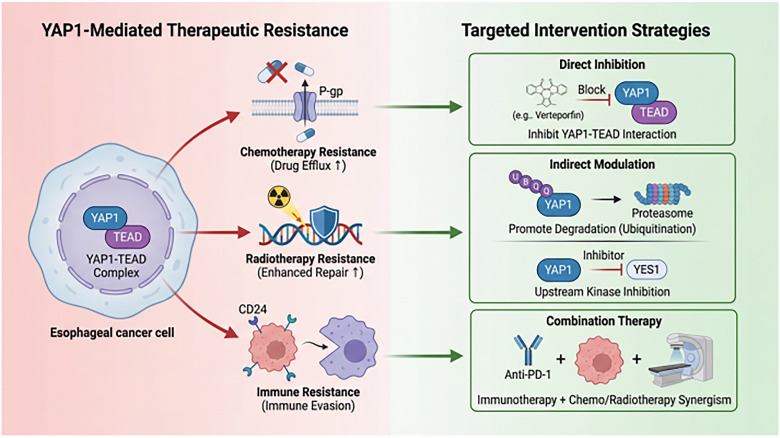
Schematic of *YAP1*-driven EC treatment resistance mechanisms and targeted intervention strategies. Abnormal activation of *YAP1* is associated with resistance to EC therapy. As illustrated, the *YAP1*-TEAD transcriptional complex within the nucleus mediates resistance to chemotherapy (e.g., by upregulating the drug efflux pump P-gp), radiotherapy (e.g., by enhancing DNA damage repair capacity), and immunotherapy (e.g., through CD24-mediated macrophage immune escape) by regulating downstream target genes. Current targeted intervention strategies primarily include: directly inhibiting *YAP1*-TEAD protein interactions; indirectly regulating *YAP1’*s upstream kinases or its protein stability to promote degradation; and combining *YAP1* inhibitors with conventional chemoradiotherapy or immune checkpoint inhibitors (e.g., anti-PD-1 antibodies). Abb: P-glycoprotein (P-gp); Programmed cell death protein 1 (PD-1).

The development of therapeutic resistance is a major clinical challenge in EC. *YAP1* may be a driver of this resistance, enabling tumor cells to survive chemotherapy, radiotherapy, and immunotherapy by regulating drug efflux, DNA repair, and the immune microenvironment. This section explores strategies to overcome resistance by targeting the *YAP1* pathway, including direct inhibition of the *YAP1*-TEAD complex, indirect intervention via upstream regulators, and novel combination therapies. These approaches aim to enhance tumor therapeutic sensitivity and improve treatment outcomes for EC patients (as shown in Fig. 5).

#### 3.6.1 Mediating Therapeutic Resistance

Abnormal activation of *YAP1* may protect tumor cells from therapeutic killing through multiple mechanisms or serve as a key factor contributing to resistance to chemotherapy, radiotherapy, and immunotherapy in esophageal carcinoma (EC).

Regarding chemotherapy resistance, *YAP1* participates in regulating the expression of various drug efflux pumps (such as P-glycoprotein), potentially contributing to the development of chemotherapy resistance in EC cells. Research indicates that restoring FAT1 expression enhances YAP phosphorylation and reverses chemotherapy resistance, suggesting the FAT1-*YAP1* axis may serve as a therapeutic target to overcome ESCC treatment resistance [13]. The *YAP1* inhibitor Verteporfin reverses IQGAP1-induced ferroptosis resistance in ESCC cells and enhances paclitaxel chemotherapy sensitivity [81].

Regarding radiation resistance, *YAP1* plays a crucial role in regulating radiotherapy resistance. *YAP1* forms a positive feedback loop with CDK6 or enhances DNA damage repair capacity by phosphorylating Rb protein, thereby diminishing radiosensitivity [5]. Xin et al. found that radiotherapy induces upregulation of CD155 expression, mediating radiation resistance through activation of the Hippo-*YAP1* pathway [82]. The interaction between *YAP1* and hypoxia-inducible factor 1-alpha (HIF-1α) jointly promotes tumor cell survival and an immunosuppressive microenvironment, leading to ESCC radiation resistance [83]. Yuan et al. revealed that inhibiting USP14 induces *YAP1* ubiquitination and degradation, accumulates DNA damage, suppresses EMT, and enhances radiation sensitivity [84].

Regarding immune resistance, preclinical evidence suggests *YAP1* may transcriptionally activate the immune checkpoint molecule CD24, aiding tumor cells in evading macrophage phagocytosis—a potential pathway for establishing an immunosuppressive microenvironment [72].

In summary, *YAP1* may contribute to the multimodal resistance of EC cells by upregulating efflux pumps, enhancing DNA repair, and shaping an immunosuppressive microenvironment. Consequently, targeting the *YAP1* signaling axis could represent a key strategy to overcome clinical resistance in EC and improve prognosis; however, its efficacy and safety require further clinical validation.

#### 3.6.2 Targeting YAP1 for EC Treatment

Given the diagnostic and prognostic potential of *YAP1*, targeting its signaling axis has emerged as a novel strategy to overcome treatment bottlenecks in EC. Current approaches include direct inhibition, indirect modulation, and combination therapies.

Direct Targeting Strategies

Direct targeting strategies aim to precisely interfere with *YAP1’*s transcriptional activity or cellular localization, primarily by blocking the formation of the *YAP1*-TEAD transcriptional complex and inhibiting *YAP1* nuclear translocation.

Blocking *YAP1*–TEAD Interaction: *In vitro* studies demonstrate that the verteporfin inhibitor suppresses angiogenesis by downregulating proangiogenic factors such as VEGFA and MMP-2. This inhibits the formation of the *YAP1*-TEAD complex, thereby suppressing angiogenesis and proliferation in endothelial cells while inducing apoptosis and enhancing chemotherapy sensitivity [85]. Furthermore, Verteporfin modulates *YAP1’*s SUMOylation and phosphorylation states, thereby influencing its intracellular localization and function [50]. *In vitro* and *in vivo* studies confirm that *YAP1*–TEAD inhibitors (e.g., TED-347) effectively suppress EC cell proliferation and migration while blocking early pulmonary metastasis [74,76]. Clinical trials revealed that the inhibitor AT-101, by targeting *YAP1*-TEAD, eliminates tumor stem cell properties conferred by the *YAP1*-SOX9 axis and enhances the efficacy of chemoradiotherapy [86].

Currently, drug development targeting the *YAP1*–TEAD axis has progressed from preclinical studies (*in vitro* and animal model validation) to early-phase clinical trials. For example, the HSP90 inhibitor AUY922 reduced tumor burden and improved immune cell infiltration in a Phase I gastric cancer trial by inhibiting the *YAP1*-TEAD pathway [42]; *YAP1*-TEAD inhibitors overcame *YAP1*/TAZ-driven resistance in a Phase II trial of KRAS G12C-mutated solid tumors [44]. These early clinical data provide preliminary support for the concept, but their efficacy and safety in EC require evaluation in specifically designed clinical trials.

Inhibiting *YAP1* Nuclear Translocation: In YES1-amplified EC models, YES1 kinase inhibitors suppressed *YAP1* nuclear translocation and transcriptional activity. By modulating the downstream *YAP1*-CREB1 axis, they reduced CD8^+^ T cell exhaustion and enhanced antitumor immunity [62].

Indirect Targeting Strategy

Indirect targeting of *YAP1* can be achieved by intervening in its upstream regulatory factors, ubiquitin modification, associated kinase activity, or interacting signaling pathways.

Genetic intervention: Knocking down *YAP1* expression via gene silencing technology suppresses the malignant phenotype of EC cells and promotes apoptosis by downregulating EGFR expression and blocking the *YAP1*-EGFR signaling axis [32].

Regulating Upstream Kinases and Ubiquitination: Targeting the oncogene RNF106 stabilizes LATS2 and enhances *YAP1* phosphorylation, offering a novel approach to indirectly inhibit *YAP1* activity [40]. Targeting USP14 promotes *YAP1* ubiquitination and degradation in ESCC cell lines, and in combination with drugs, significantly delays tumor growth in patient-derived xenograft (PDX) models [84].

Blocking Associated Signaling Pathways: The interaction between metabolic reprogramming and *YAP1* signaling has also emerged as a therapeutic target. Chen et al. proposed a dual FGFR2/IL-8 blockade strategy to overcome ESCC treatment resistance by indirectly modulating necrosis-dependent *YAP1* signaling [65]. Novel YES1/SRC inhibitors induce tumor regression in xenograft models by blocking *YAP1* nuclear localization and abnormal Hippo pathway activation [87]. The microtubule-associated protein CKAP5 promotes YAP nuclear translocation by stabilizing microtubules in ESCC; targeting the CKAP5-YAP axis may reverse tumor progression and cisplatin resistance [88].

Exploration of Combination Therapy

Given the redundancy and compensatory nature of the *YAP1* signaling pathway, combination therapy strategies are crucial for achieving sustained efficacy. The core approach involves combining *YAP1* inhibitors with existing standard therapies or other targeted pathways to generate synergistic or sensitizing effects.

Combination with radiotherapy: Regarding chemotherapy sensitization, silencing *YAP1* enhances the cytotoxic effect of cisplatin on ESCC cells by activating the Hippo pathway [73]. CD155 mediates radiotherapy resistance through the Hippo-*YAP1* pathway, and its overexpression predicts shorter radiotherapy-survival time in ESCC patients [82].

In combination with immunotherapy: Targeting the *YAP1*-CD24 axis reverses tumor-associated macrophage-mediated immune evasion. Combining *YAP1* inhibitors with PD-1/PD-L1 blockade improves survival in tumor-bearing mice [72]. Preliminary clinical trials indicate that, calcium electroporation technology modulates the EC microenvironment by regulating gene expression of CXCL14, CCL21, and others, indirectly influencing *YAP1* pathway activity. Preliminary clinical trials indicate it can remodel immune cell infiltration [89].

These preliminary preclinical and early clinical studies (currently dominated by ESCC data) offer proof-of-concept and experimental rationale for *YAP1*-targeted EC therapy, though most mechanisms remain hypothetical (as shown in Table 3). Direct targeting of *YAP1*-TEAD interactions, indirect regulation of upstream factors, and combination therapy strategies show promising application prospects. Nevertheless, translating these approaches into clinical practice faces significant challenges that require urgent resolution and further in-depth investigation.

**Table 3:** YAP1-mediated therapeutic resistance mechanisms and targeting strategies.

Category	Core Mechanism	Functional Overview and Representative Agents	References
**Therapeutic Resistance**			
Chemotherapy Resistance	Inhibition of Ferroptosis	Enhanced drug efflux; Verteporfin can reverse	[13,81,88]
Radiotherapy resistance	CDK6/*YAP1* positive feedback, CD155/Hippo-YAP pathway, HIF-1α interaction	Enhances DNA repair, inhibits apoptosis; USP14 inhibitors may sensitize	[5,82,84]
Immune resistance	*YAP1* transcription activates CD24	Mediates macrophage immune evasion; Targeting *YAP1*-CD24 axis	[72]
**Targeting Strategies**			
Direct inhibition	Blocking *YAP1*-TEAD interaction, inhibiting nuclear translocation	Verteporfin, AT-101, TED-347, YES1 inhibitors	[62,74,85,86]
Indirect regulation	Targeting upstream factors such as RNF106 and USP1; Blocking pathways like FGFR2/IL-8	Promotes *YAP1* degradation; FGFR2/IL-8 bispecific antibody	[40,65,84]
Combination Therapy	Combined with chemotherapy, radiotherapy, or immune checkpoint inhibitors	Reversing drug resistance; *YAP1* inhibitor combined with PD-1/PD-L1 blockade	[72,73,81]

Note: TEA domain (TEAD); Ring finger protein 106 (RNF106); Ubiquitin-specific-processing protease 1 (USP1); Fibroblast growth factor receptor 2 (FGFR2); Interleukin-8 (IL-8); Programmed cell death protein 1 (PD-1); Programmed death-ligand 1 (PD-L1).

## 4 Discussion

### 4.1 Research Challenges

**Figure 6:**
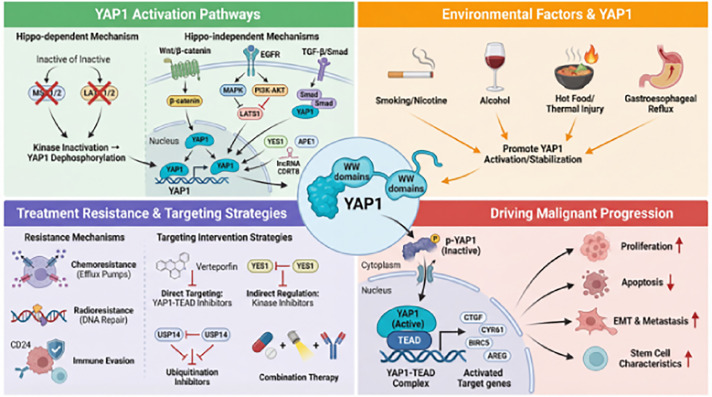
*YAP1* drives malignant progression and therapeutic resistance in EC by integrating upstream environmental factors, inflammatory signals, and metabolic reprogramming. The left panel illustrates three major input signals activating *YAP1* via Hippo-dependent or -independent pathways. Activated *YAP1* undergoes dephosphorylation and translocates to the nucleus, where it binds to TEAD transcription factors to regulate downstream target gene expression. The right panel illustrates two major outputs following *YAP1* activation: promoting malignant tumor biology (such as proliferation, EMT, and stemness maintenance) and mediating resistance to chemotherapy, radiotherapy, and immunotherapy. Abb: tryptophan-tryptophan domain (WW domain).

Despite *YAP1’*s promising potential in EC (as shown in Fig. 6), it currently faces multiple significant challenges. These challenges stem not only from the complexity of their molecular mechanisms, but also from the disconnect between existing research in mechanism elucidation, model development, and clinical validation.

#### 4.1.1 The Challenge of Heterogeneity of EC Subtypes

The specific functions and regulatory mechanisms of *YAP1* in different subtypes of EC remain incompletely understood. Significant differences exist in the molecular mechanisms, clinical relevance, and targeting strategies between ESCC and EAC. However, most mechanism studies have focused on ESCC, with insufficient data on EAC and a lack of multicenter validation, posing risks of extrapolation beyond the specific subtype (as shown in Table 4).

**Table 4:** Summary of key studies on *YAP1* in EC.

Subtype	Model	Key Findings and Implications	Reference
ESCC	Cell Lines, Clinical Samples	OTUB2 deubiquitinates and stabilizes *YAP1*/TAZ, promoting cell proliferation and migration	[12]
ESCC	Cell lines, animal models	FAT1/PTPN14 deficiency leads to LATS2 degradation, activating *YAP1* to promote progression and drug resistance	[13]
ESCC	Clinical Samples, *In Vitro* Experiments	Ezrin overexpression positively correlates with *YAP1* levels, promoting cell proliferation and invasion	[14]
ESCC	Cell lines	USP36 deubiquitinates and stabilizes *YAP1*, enhancing oncogenic activity	[16]
ESCC	Cell lines	RGS16 disrupts MST1-LATS1 interaction, promoting *YAP1* activation and migration	[39]
ESCC	Cell Line	RNF106 degrades LATS2 to release *YAP1* inhibition, promoting EMT and metastasis	[40]
ESCC	Clinical Samples	SQLE amplification promotes YAP nuclear translocation and activates anti-apoptotic genes	[46]
ESCC	Cell lines	C12orf59 overexpression activates YAP, inducing EMT transcription factor expression	[47]
ESCC	Cell Line	AJUBA regulates YAP/TAZ nuclear localization, promoting proliferation and migration	[48]
ESCC	Clinical Samples, Animal Models	YAP-CD24 axis mediates immune evasion and reduces macrophage phagocytosis	[72]
ESCC	Cell lines	YAP-TEAD complex upregulates IRS2, accelerating cell cycle progression	[73]
ESCC	Cell Line	TEAD4-YAP interaction promotes proliferation and invasion; its inhibition suppresses malignant phenotypes	[74]
ESCC	Animal Model	GPRC5A relieves YAP inhibition, mediating EMT-like phenotype to drive lung metastasis	[76]
ESCC	Cell Line	LncRNA KTN1-AS1 activates YAP signaling and promotes tumor progression	[78]
ESCC	Clinical Cohort	*YAP1* amplification predicts poor prognosis with concurrent chemoradiotherapy	[80]
ESCC	Cell Line	Verteporfin Inhibits YAP-TEAD Interaction, Reverses Chemotherapy Resistance	[81]
ESCC	Cell Line	CD155 mediates radiotherapy resistance through the Hippo-YAP axis	[82]
ESCC	Cell Line	USP14 inhibitors promote *YAP1* degradation and enhance radiotherapy sensitivity	[84]
ESCC	Cell Line	CKAP5 stabilizes microtubules to promote YAP nuclear translocation, driving cisplatin resistance and progression	[88]
EAC	Barrett’s esophagus cell line	APE1 redox function inhibits YAP ubiquitination and degradation, promoting malignant transformation	[63]

Note: OTU deubiquitinating enzyme 2 (OTUB2); protein tyrosine phosphatase non-receptor type 14 (PTPN14); squalene epoxidase (SQLE); AJUBA LIM protein (AJUBA); insulin receptor substrate 2 (IRS2); G protein-coupled receptor class C group 5 member A (GPRC5A); cytoskeleton associated protein 5 (CKAP5).

Current understanding of *YAP1* mechanisms exhibits significant gaps in evidence. In ESCC, extensive *in vitro* and *in vivo* (animal model) studies have identified multiple potential oncogenic pathways. However, the direct causal relationship between these preclinical findings and clinical trial outcomes in ESCC patients remains to be established, and their clinical translational value requires validation through prospective interventional trials. In EAC, even systematic preclinical research is extremely scarce, let alone clinical evidence, resulting in virtually no targeted strategies for this subtype. Clinical data indicate that *YAP1* overexpression in ESCC patients is negatively correlated with CD8^+^ T cell infiltration [15], but this immune regulatory role remains a hypothesis in EAC without clinical validation. YES1 amplification, frequently observed in ESCC, directly activates *YAP1* [55], whereas EAC more commonly exhibits HER2 or TP53 mutations [90]. The causal relationship between these alterations in clinical patients requires further validation through cohort studies.

Regarding clinical translation, *YAP1*-targeted therapies developed for ESCC may not be applicable to EAC. Due to distinct driver events and microenvironmental characteristics (e.g., anatomical specificity of the gastroesophageal junction) in EAC, existing strategies lack EAC-specific validation. Their efficacy and safety require separate evaluation in future studies. Zhou et al. demonstrated that the YAP-CD24 axis mediates immune evasion in ESCC [72], whereas YAP regulation in EAC may involve distinct signaling networks. Targeting strategies have primarily been developed based on ESCC models, with limited research on EAC-specific driver mechanisms. Consequently, therapeutic approaches for EAC remain reliant on conventional regimens [91].

This subtype discrepancy reflects a deeper issue: Does current research overemphasize ESCC models, thereby overlooking EAC’s unique pathogenesis and microenvironment? This directly leads to three critical shortcomings: ① The mechanism of YAP activation in EAC remains unexplained, with existing conclusions largely extrapolated from ESCC; ② No multi-center validated biomarkers specific to EAC exist, resulting in a lack of molecular basis for clinical decision-making; ③ Regulation of *YAP1* by the immune microenvironment exhibits subtype specificity, yet the underlying causal pathways require systematic investigation.

#### 4.1.2 YAP1 Functional Duality Controversy

The function of *YAP1* in epithelial cells exhibits significant duality, potentially acting as an oncogenic factor to promote tumor progression while also exerting tumor-suppressive effects under specific conditions. The molecular mechanisms underlying this contradictory role remain incompletely elucidated (as shown in Table 5). Most studies support the pro-cancerous function of *YAP1* in ESCC (e.g., mediating EMT, immune evasion, and promoting invasive growth). However, a few studies report that *YAP1* may exert tumor-suppressing functions in long-term survivors, suggesting its role may be stage-dependent. Single-cell sequencing reveals *YAP1* function exhibits microenvironmental dependence: within tumor cores, *YAP1* promotes proliferation; at invasion fronts, it induces apoptosis via PML [33]; and high *YAP1* expression correlates with better patient prognosis [34].

**Table 5:** Key studies comparing *YAP1* dual functionality in EC.

Functional Orientation	Model/System	Key Findings and Evidence	Reference
**Oncogenic**	ESCC cell lines, clinical samples	OTUB2 deubiquitinates and stabilizes *YAP1*/TAZ, promoting cell proliferation and migration	[12]
**Oncogenic**	ESCC cell lines, animal models	FAT1/PTPN14 deficiency leads to LATS2 degradation, *YAP1* activation promotes progression and cisplatin resistance	[13]
**Oncogenic**	ESCC clinical samples, *in vitro* experiments	Ezrin overexpression positively correlates with *YAP1* levels, promoting proliferation and invasion	[14]
**Carcinogenic**	ESCC cell lines	USP36 deubiquitinates and stabilizes *YAP1*, enhancing oncogenic activity	[16]
**Carcinogenic**	ESCC cell lines	RNF106 promotes EMT and metastasis by degrading LATS2 and relieving *YAP1* inhibition	[40]
**Carcinogenic**	ESCC Clinical Samples	SQLE amplification promotes YAP nuclear translocation and activates anti-apoptotic genes	[46]
**Carcinogenic**	ESCC clinical samples, animal models	*YAP1*-CD24 axis mediates immune evasion and reduces macrophage phagocytosis	[72]
**Carcinogenic**	ESCC cell lines	*YAP1*-TEAD complex upregulates IRS2, accelerating cell cycle progression	[73]
**Carcinogenic**	ESCC cell lines	TEAD4 interacts with *YAP1* to promote ESCC cell proliferation, migration, and invasion,	[74]
**Carcinogenic**	Esophageal *In Situ*/Lung Metastasis Model	GPRC5A relieves YAP inhibition, mediating EMT-driven lung metastasis	[76]
**Tumor Suppression**	Hepatic Resection Animal Model	*YAP1* activation suppresses colorectal cancer liver metastasis by regulating glutamine metabolism	[27]
**Tumor Suppression**	Multi-cancer cell line studies	*YAP1* promotes ferroptosis to inhibit cell growth	[28]
**Tumor Suppression**	ESCC Clinical Samples	*YAP1* regulates oncogenic factor TAZ, whose overexpression correlates with longer OS in patients	[33]
**Tumor Suppressor**	ESCC Cohort (TCGA Database)	High *YAP1* expression correlates with better patient prognosis	[34]
**Tumor Suppression**	ESCC Clinical Samples	Among long-term survivors, *YAP1*-positive patients demonstrated significantly prolonged DFS and OS	[35]

Note: overall survival (OS); disease-free survival (DFS).

Existing studies have largely remained at the level of observational phenomena, lacking systematic analysis of spatiotemporal dynamics and microenvironmental influences. Functional reversals may occur between early-stage and late-stage tumors. The inconsistencies in existing findings suggest that *YAP1* cannot be simplistically categorized as either an oncogene or a tumor suppressor gene. Instead, we must investigate the molecular switches governing its functional transitions. This spatiotemporal functional shift underscores the urgent need to validate and explore the microenvironmental signals and molecular switches underlying *YAP1’*s dual roles through methods such as single-cell multi-omics, thereby testing the stage-dependent hypothesis.

#### 4.1.3 Mechanistic Complexity and Resistance Challenges in Targeted Therapy

The clinical translation of *YAP1*-targeted therapies faces multiple obstacles, including significant challenges such as complex mechanisms, redundant signaling pathways, activation of compensatory pathways, and potential off-target effects. Existing drugs generally lack systematic evaluation of these factors, making single-strategy approaches highly susceptible to failure in clinical trials. Simultaneously, off-target effects of certain inhibitors may activate novel oncogenic signaling pathways—such as verteporfin inducing Wnt/β-catenin activation—underscoring the necessity of incorporating cross-pathway off-target risk analysis into new drug design.

First, existing *YAP1* inhibitors remain primarily in the preclinical research stage, lacking human safety data. Second, compensatory mechanisms within the *YAP1* signaling pathway may lead to treatment failure with monotherapy, necessitating exploration of combination regimens. Finally, targeting *YAP1* may interfere with its physiological functions in normal tissues, and potential toxicity risks urgently require resolution through more precise delivery systems.

Targeting the *YAP1*-TEAD complex demonstrates therapeutic potential in preclinical models, though tissue-specific effects warrant attention [3]. High *YAP1* expression correlates with PD-1 inhibitor resistance, and evidence suggests it may synergistically regulate tumor stem cell properties with TEAD3 [31]. Existing targeted drugs (e.g., Verteporfin) exhibit severe off-target effects. Their inhibition of angiogenesis concurrently activates the Wnt/β-catenin pathway, potentially inducing metastasis risks. Additionally, phototoxicity issues limit their clinical application. Risks associated with YES1 kinase inhibitors include potential impairment of normal SRC family kinase function, necessitating clinical monitoring for cardiovascular and immune-related adverse events [87].

Targeted *YAP1* strategies show great promise. However, these approaches face challenges in clinical translation, including pharmacokinetic optimization, control of off-target effects, and tissue-specific delivery. They may increase the risk of immune-related adverse events, and efficacy differences across patients with distinct immune phenotypes remain unclear. Furthermore, most drugs remain in preclinical or early clinical stages, lacking long-term safety and efficacy data.

#### 4.1.4 Insufficient Knowledge of the Tumor Microenvironment and Immune Escape

The regulatory role of *YAP1* in the EC microenvironment remains largely at the level of association, lacking in-depth causal and mechanistic analysis. Existing studies predominantly focus on tumor cells themselves, overlooking the regulation of *YAP1* activity by immune cells, stromal cells, and others. Whether *YAP1* directly suppresses T cell function or indirectly creates an immunosuppressive environment by remodeling stromal cells remains unknown. *YAP1* activity is modulated by cytokines secreted by tumor-associated macrophages, potentially exhibiting distinct patterns across different tumor progression stages. Moreover, the spatiotemporal dynamics of *YAP1* expression within the tumor microenvironment are challenging to accurately capture, complicating biomarker validation.

The synergistic interaction between *YAP1* and immune checkpoints remains poorly understood. Most related reports are limited to correlation analysis rather than causal validation, failing to clarify whether *YAP1* directly regulates the expression of immune checkpoint molecules such as PD-L1. Furthermore, the mechanisms by which *YAP1* influences antigen presentation and T cell functional exhaustion remain unexplained. This lack of understanding limits the feasibility assessment of incorporating *YAP1* into combination immunotherapy strategies.

#### 4.1.5 Spatial and Temporal Heterogeneity of Molecular Mechanisms and Modeling Limitations

Key unanswered questions remain at the molecular mechanism level: The spatiotemporal dynamic heterogeneity of the *YAP1* regulatory network and its interactions with multiple signaling pathways remain poorly understood, and existing models struggle to accurately reflect the complexity of human tumors. There is a lack of systematic screening for *YAP1* fusion gene events, the mechanism by which *YAP1* interacts with other key signaling pathways is unclear, and how metabolic reprogramming affects the *YAP1*-dependent immune microenvironment has not been elucidated.

The primary obstacle is the lack of genetic animal models that mimic the evolution of human EC and preclinical models of treatment response, which limits the translational value of preclinical data. Existing studies have relied mostly on xenograft models, whose tumor microenvironments are significantly different from those of the human body, making it difficult to fully reproduce tumor heterogeneity and the dynamic regulatory network of the *YAP1* signaling pathway.

#### 4.1.6 Standardization Challenges for YAP1 Detection Platforms

The clinical application of *YAP1* as a biomarker still faces challenges such as inconsistent detection standards and insufficient dynamic monitoring capabilities. Significant variations in testing methods and interpretation criteria across different laboratories compromise the comparability of results. Although studies confirm that *YAP1* nuclear localization is associated with EC radiotherapy resistance, chemotherapy resistance, and poor prognosis, standardized biomarker detection protocols and multicenter validation data are lacking. Unified assessment standards are absent for dynamically changing *YAP1* expression levels, detection standardization, and analyses of its association with immune cells in the microenvironment [79].

Current *YAP1* detection primarily relies on immunohistochemistry, Western blot, qPCR, and multi-omics analysis, yet significant shortcomings exist in sensitivity, specificity, dynamic monitoring capability, and clinical scalability. Inconsistent detection standards across laboratories compromise result comparability. The absence of real-time detection technologies for *YAP1* activity states (e.g., phosphorylation, nuclear localization) limits its application in predicting treatment responses. Furthermore, liquid biopsy platforms remain exploratory, with methods like exosomal *YAP1* protein and circulating tumor cell nuclear localization imaging lacking multicenter validation, hindering the establishment of standardized detection systems.

### 4.2 Future Research Directions

To address these challenges, future research should focus on the following priorities:

#### 4.2.1 Conducting Subtype-Specific Mechanism and Therapeutic Studies

Improving molecular typing on the basis of *YAP1* activity status to guide precise intervention. To elucidate the mechanism of action of *YAP1* in EAC, EAC-specific genetically engineered mouse models or organoids to mimic EAC have been established, and the function and regulatory network of *YAP1* in these models have been systematically analyzed to formulate subtype-based stratified therapeutic strategies.

#### 4.2.2 Analyzing the Mechanism of Functional Heterogeneity

Elucidating the molecular basis of *YAP1* functional heterogeneity is key to overcoming the challenges of targeted therapy. What microenvironmental signals or intrinsic tumor factors determine *YAP1’*s functional transition from a potential early-stage “guardian” to a late-stage “driver”?

Single-cell multi-omics and spatial transcriptomics technologies should be employed to decipher how microenvironmental factors dynamically regulate *YAP1* function, thereby validating stage-dependent hypotheses. Constructing a functional state map of *YAP1* will precisely delineate its molecular switches and microenvironmental conditions governing oncogenic or tumor-suppressive roles, providing theoretical foundations and screening platforms for targeted drug development. Resolving these contradictory findings requires systematic analysis of multiple mechanisms: ① Stage dependency: *YAP1* may suppress malignant transformation in early tumors while driving progression in advanced stages; ② Microenvironmental regulation: Matrix stiffness or inflammatory factors influence *YAP1* nuclear translocation; ③ Isoform differences: distinct *YAP1* splicing variants may exhibit opposing functions; ④ Post-translational modifications: dynamic regulation of *YAP1* activity through phosphorylation, ubiquitination, and other modifications. ⑤ Furthermore, targeting *YAP1* therapeutically requires caution: in scenarios where *YAP1* exerts tumor-suppressing effects (e.g., early-stage lesions), its inhibition may exert harmful effects and paradoxically promote tumor progression.

#### 4.2.3 Developing Novel Targeting Strategies

For the development and optimization of targeted drugs, first, highly selective and low-toxicity inhibitors of *YAP1*-TEAD protein interactions are developed, blocking the formation of the transcription complex. To address the existing phototoxicity problem, nonphotosensitive drugs need to be designed; second, highly selective inhibitors targeting upstream kinases (e.g., YES1/SRC) need to be developed; and third, the use of nanotechnology to improve the tumor-targeting ability of the drug and reduce systemic toxicity.

Innovative combination therapeutic strategies are the focus of future research, with the core goal of synergistically blocking the main pathway and compensatory bypass to overcome microenvironment-mediated protection. We focused on overcoming the bottleneck of *YAP1*-mediated resistance to radiotherapy and verified the synergistic effect of *YAP1* inhibitors combined with radiotherapy, CDK4/6 inhibitors or immune checkpoints.

#### 4.2.4 Deep Integration of Multidimensional Mechanisms

Genomics, transcriptomics, epigenomics, proteomics and spatial technologies need to be integrated for joint analysis, with the core focus on resolving the following: ① the dynamic heterogeneity of *YAP1* activity in the tumor space (*in situ*, invasive front, metastatic foci) and time (before and after treatment, recurrence evolution); and ② how *YAP1* interacts with metabolism and immune cells in the EC microenvironment to form a feedback loop, which together drives malignant progression and treatment resistance.

#### 4.2.5 Innovative Modeling

Develop more precise genetic engineering models. For example, models of conditional knockdown or activation of *YAP1* were used to observe the role of *YAP1* in a specific time and space [75]. The establishment of organoid models can simulate the real tumor microenvironment and observe the behaviors of tumor cells, such as growth, migration and drug response, *in vitro*.

AI prediction models can also be developed to construct *YAP1*-based efficacy prediction algorithms to guide the tiered application of targeted drugs. For example, by analyzing the structure and bioactivity data of existing drugs, AI can help identify potential *YAP1* inhibitors [80], which can accelerate the development of new drugs and provide new hope for the treatment of EC and other diseases.

#### 4.2.6 Developing Future Detection Platforms and Exploring Auxiliary Strategies

Future efforts should focus on developing highly sensitive, quantitative, and dynamically monitorable *YAP1* detection platforms. These platforms should integrate single-cell multi-omics and spatial transcriptomics technologies to achieve precise capture of *YAP1* activity states. In the liquid biopsy domain, approaches such as ctDNA detection of *YAP1* fusions/amplifications/mutations, analysis of *YAP1* protein phosphorylation status in exosomes, and imaging of circulating tumor cell nuclear localization should be explored, alongside establishing a multicenter standardized validation system. Artificial intelligence can be leveraged for multimodal data integration to construct *YAP1* activity prediction models, aiding clinical stratification and efficacy assessment. Concurrently, coordinated development of detection platforms and therapeutic strategies should be advanced to achieve integrated detection-intervention pathways.

Explore nutritional or metabolic interventions to develop low-toxicity adjuvant strategies to increase the sensitivity of existing therapies or for prevention: Investigate the effects of specific nutrients (e.g., selenium, VD) or metabolic modulators on *YAP1* nuclear translocation and activity.

#### 4.2.7 Enhancing Cross-Cancer Borrowing and Translation

Accelerate the development of EC-targeted therapies by borrowing research results and lessons learned from other cancer types that target *YAP1* or related pathways. Develop combination therapies targeting EC stromal sclerosis by learning from the experience of stromal-targeted therapies in hepatocellular carcinoma. In the strategy of deubiquitination regulation in ovarian cancer, deubiquitinating enzyme inhibitors such as USP7/UBE2T are used to block the stabilizing effect of USP36 on *YAP1* and overcome drug resistance. Drawing from colorectal cancer research, metabolites of *Lactobacillus plantarum* K25 (such as (Z)-18-Octadec-9-enolide) can stably bind to aquaporin 8 (AQP8) through molecular docking, thereby influencing oxidative stress and migration in tumor cells [92]. Such studies provide a reference direction for exploring interactions between gut microbiota metabolites and membrane proteins in EC, which may regulate *YAP1* or related pathways.

The application of advanced models, biomarker development experience, and combination strategy innovations in other cancer types can accelerate the development of *YAP1*-targeted therapies in EC.

#### 4.2.8 Clinical Translation and Biomarker Validation

Based on this systematic analysis of *YAP1’*s multidimensional regulatory network and therapeutic resistance mechanisms in EC, the following priority recommendations are proposed for its clinical application:

Standardization of immunohistochemical scoring: Establish a unified *YAP1* immunohistochemical scoring system with defined criteria for staining intensity, proportion of positive cells, and nuclear localization to ensure comparability of cross-center test results. ② Prospective Validation Cohort: Conduct multicenter, large-sample prospective clinical cohort studies to validate the stability and sensitivity of *YAP1* and its downstream molecules in diagnosis, prognostic assessment, and treatment response prediction. ③ Companion Diagnostic Development: Simultaneously develop companion diagnostic platforms with clinical trials to enable real-time detection of *YAP1* activity states (e.g., phosphorylation, nuclear localization), guiding patient selection and efficacy monitoring. ④ Endpoint Selection: In *YAP1*-targeted or combination therapy trials, prioritize endpoints such as OS, PFS, and degree of immune microenvironment improvement, while concurrently collecting biomarker data.

## 5 Summary and Outlook

The malignant progression of EC involves the interplay of multiple factors. As a core regulatory factor, *YAP1* possesses dual potential for both oncogenic and tumor-suppressive functions, playing a pivotal role in multidimensional signaling networks. This review comprehensively examines its mechanisms, clinical value, and subtype differences in EC, while highlighting the distinctions between ESCC and EAC in molecular mechanisms, clinical relevance, and targeting strategies. *YAP1* regulates downstream genes through both Hippo-dependent and Hippo-independent mechanisms, and is associated with phenotypic changes such as tumor cell proliferation, apoptosis inhibition, EMT, and drug resistance. Its clinical value as a potential prognostic marker and therapeutic target underscores the theoretical basis for *YAP1*-guided precision medicine.

Despite promising prospects, *YAP1*-targeted therapy faces challenges, including complex mechanisms, subtype differences, resistance risks, safety concerns, and insufficient biomarker validation. These factors constrain clinical adoption, necessitating multidisciplinary collaboration and cross-cancer insights. Future priorities should include: (1) elucidating dynamic shifts in *YAP1* function across tumor stages and microenvironments; (2) Developing subtype-specific targeted drugs and combination therapy strategies; (3) Establishing standardized detection platforms to validate biomarkers; (4) Integrating cross-cancer insights to accelerate clinical translation. Through multidisciplinary collaboration, we can construct a personalized precision treatment system based on the *YAP1* signaling network, improve EC patient outcomes, and provide new opportunities for the advancement of precision medicine.

## Data Availability

Not applicable.
